# Supplementary material for: NV center based nano-NMR enhanced by deep learning
Source: Sci Rep. 2019 Nov 28;9:17802. doi: 10.1038/s41598-019-54119-9 (PMC6882844; doi:10.1038/s41598-019-54119-9)
Supplement: Supplementary file 1 — Supplementary Infromation [file 41598_2019_54119_MOESM1_ESM.pdf]

Supplementary Material for NV center based nano-NMR enhanced by deep learning

Nati Aharon,[1] Amit Rotem,[1] Liam P. McGuinness,[2] Fedor Jelezko,[2] Alex Retzker,[1] and Zohar Ringel[1]

[1]*Racah Institute of Physics, The Hebrew University of Jerusalem, Jerusalem 91904, Givat Ram, Israel*
[2]*Institute for Quantum Optics, Ulm University, Albert-Einstein-Allee 11, Ulm 89081, Germany*
(Dated: June 5, 2019)

The compressed file "DLNMR.zip" contains the following code and data files:

1. Discrimination1.py - This Python file includes the code which generates the data for Fig. 4 and Fig. 5 of the manuscript.
2. Discrimination2.py - This Python file includes the code which generates the data for Fig. 6 of the manuscript based on the numerical model.
3. Discrimination3.py - This Python file includes the code which generates the data for Fig. 6 of the manuscript based on the experimental data.
4. Resolution1.py - This Python file includes the code which generates the data for $P_{M_{DL}}$ and $P_{M_{corr}}$ in Fig. 8 of the manuscript.
5. Resolution2.nb - This Mathematica file includes the code which generates the data for $P_{M_{FB}}$ in Fig. 8 of the manuscript.
6. Data1.txt - This file includes the experimental measurement results of the AC signal with a frequency of $\omega_1 = 250$ Hz.
7. Data2.txt - This file includes the experimental measurement results of the AC signal with a frequency of $\omega_2 = 251.6$ Hz.
8. ResData1.dat - This file includes numerically generated data for a single-frequency signal, which is used in Resolution.py.
9. ResData0d3.dat, ResData0d5.dat, ResData0d7.dat, ResData0d9.dat, ResData1d1.dat, and ResData1d3.dat - These files include the numerically generated data for two-frequency signals with $\Delta = 0.3\frac{2\pi}{T_2}$, $\Delta = 0.5\frac{2\pi}{T_2}$, $\Delta = 0.7\frac{2\pi}{T_2}$, $\Delta = 0.9\frac{2\pi}{T_2}$, $\Delta = 1.1\frac{2\pi}{T_2}$, and $\Delta = 1.3\frac{2\pi}{T_2}$ respectively. This data is used in Resolution.py
